# Personality Prediction with Hybrid Genetic Programming using Portable EEG Device

**DOI:** 10.1155/2022/4867630

**Published:** 2022-06-01

**Authors:** Harshit Bhardwaj, Pradeep Tomar, Aditi Sakalle, Maneesha Sakalle, Rishi Asthana, Arpit Bhardwaj, Wubshet Ibrahim

**Affiliations:** ^1^Department of CSE, USICT, Gautam Buddha University, Greater Noida, India; ^2^Department of Mathematics, Govt. S. N. P. G. College, Khandwa, India; ^3^Department of Applied Sciences, SoET, BML Munjal University, Gurugram, India; ^4^Department of CSE, BML Munjal University, Gurugram, India; ^5^Department of Mathematics, Ambo University, Ambo, Ethiopia

## Abstract

This work suggests a method to identify personality traits regarding the targeted film clips in real-time. Such film clips elicit feelings in people while capturing their brain impulses using the electroencephalogram (EEG) devices and examining personality traits. The Myers–Briggs Type Indicator (MBTI) paradigm for determining personality is employed in this study. The fast Fourier transform (FFT) approach is used for feature extraction, and we have used hybrid genetic programming (HGP) for EEG data classification. We used a single-channel NeuroSky MindWave 2 dry electrode unit to obtain the EEG data. In order to collect the data, thirty Hindi and English video clips were placed in a conventional database. Fifty people volunteered to participate in this study and willingly provided brain signals. Using this dataset, we have generated four two-class HGP classifiers (HGP1, HGP2, HGP3, and HGP4), one for each group of MBTI traits overall classification accuracy of the HGP classifier as 82.25% for 10-fold cross-validation partition.

## 1. Introduction

The word personality is originated from the word persona, relating to the mask used in the theater by the performers [1]. Early theories suggested that in the physical appearance of men, personality was conveyed. The theory of evaluating personality by measuring the patterns of bumping people's skull was an early method founded by Franz Joseph Gall, a German scientist named phrenology [2]. However, since the rigorous experimental study did not confirm the theory's assumptions, phrenology is generally debunked in contemporary psychology. The psychologist, William Herbert Sheldon, advocated another method, known as somatology [3], which was focused on the idea that we might distinguish an individual from body types of individuals.

As with phrenology, the theory's findings have not been confirmed by the experimental study, and somatological psychology has now been disapproved. Another method is regarded as physiognomy of personality identification [4], using which face traits can be measured. Contrary to phrenology and somatology, for which no evidence of science is available, contemporary science has shown that certain facets of an individual character can be identified in abundance by looking at their face alone. It is not easy to distinguish personality from the face without these results [5]. In the end, the physiognomy predictions seem to find no empirical support. After the failure of all the above approaches, another approach known as personality traits [6] was discovered. In this approach, the personality is characterized by relatively resilient traits and affects our actions in many situations.

Trait psychology is based on the concept that people differ in their status based on a set of essential qualities that persist over time and circumstances. There were several models proposed for determining a person's personality traits. The Big Five-Factor (BFF) model [7, 8] and Myers–Briggs Type Indicator (MBTI) model [9] are the popular models of predicting personality. The BFF theory includes five traits, i.e., OCEAN [10]. Openness: a person's willingness to consider different items; conscientiousness implies individuals who are coordinated, committed, and who are planning; extroversion shows anxious people, engaged, talking, and enthusiastic; agreeableness shows the friendliness amongst people; neuroticism relates to control over the emotions. The MBTI model consists of four dimensions of personality, and each dimension consists of two traits in versus. Therefore, the MBTI model contains eight traits in total, i.e., extraversion (E) vs. introversion (I) indicates where and how you get your energy, sensing (S) vs. intuition (I) indicates how you take in information, thinking(T) vs. feeling(F) indicates how you make decisions, and judging(J) vs. perceiving(P) indicates how do you prefer to live your life every day. Researchers can use the physiological signal to obtain a greater understanding of the individual's actions during the research. Physiological signals are far more efficient than digital footprints for recognizing personality since they provide a better degree of classification accuracy [11].

**Figure 1:**
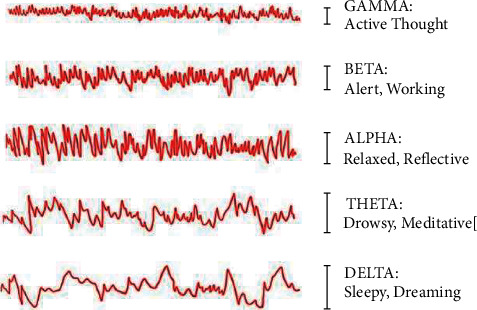
Brain wave frequency bands.

Signals from the pulse rate [12] and heart rate calculated by electrocardiogram (ECG) [13], blood pressure [14], and brain signals recorded by using electroencephalograph (EEG) [15, 16] in this group are recorded. The human brain generates physiological signals, which have grown in prominence in recent years since it is impossible to mimic brain activity using EEG signals [17]. Researchers can determine personality traits with a high degree of accuracy using EEG data [18, 19]. EEG signals record electrical activity produced by the neurons in the brains, and they have been used widely to analyze the functional changes in the brain (Imah, Rahmawati et al., 2019) [20]. Due to its different characteristics when engaging with an emotion, EEG is thought to be the most appropriate approach to record data in multiple modalities [21, 22]. EEG is a nonintrusive, quick, and cost-effective approach that makes it a favorite way of testing the brain's reactions to feelings targeting personality trait stimuli [23]. EEG signals frequency varies from 0.5 Hz to 100 Hz and are grouped into five bands: delta, theta, alpha, beta, and gamma, as shown in Figure 1, and all the bands have different frequencies. The band 0.5 Hz–50 Hz is used for the study of human brain actions in this research work. A two-stage method of extracting and classifying features is a study of EEG signals. The specific standard techniques used to extract key features from the raw EEG signals are the fast Fourier transform [24], eigenvectors [25], the wavelet transform (WT) [26], time-frequency distributions [27], empirical mode decomposition (EMD) [28], and local discriminant bases [29]. This work includes fast Fourier transform (FFT) for feature extraction, out of all the above techniques.

This paper uses FFT for feature extraction. Compared to other signal processing techniques, FFT reduces the computation time [30]. In response to movie clips that target MBTI's model traits, this study introduces a new personality model that uses hybrid genetic programming. Therefore, this study proposes a novel model for predicting personality traits dependent on hybrid genetic programming. Comparisons are made with state-of-the-art approaches [31]. Evaluation of our model is often carried out using the confusion matrix. The findings demonstrate that our approach beats all the state-of-the-art classification accuracy approaches and is a good way of trait identification through brain signals. As far as we are conscious, no one has predicted a person's personality using EEG signals by showing video clips targeting personality traits.In the remaining paper, Section 2 presents the background of FFT and some GP basic concepts.  Section 3 discusses the method overview used in this model. The discussion on the results generated is discussed in Section 4.  Section 5 concludes our research and its relevance and points out the future work and scope of our paper.

## 2. Background

This section describes the key context for this method, namely FFT, for the extraction of features and GP life cycle. The prediction of personality traits can be performed by classifying the received EEG signals in the frequency bands containing EEG signals based on features and patterns. The points mentioned below are the steps to be followed for implementing a real-time system for predicting personality.

- EEG signals are extracted using the NeuroSky MindWave Mobile 2 when the participants are watching film clips.
- Preprocessing of EEG signals derived via FFT.
- Use of HGP for classification.

### 2.1. Fast Fourier Transform

The extraction of significant EEG signal characteristics is the initial step in successfully classifying personality traits. EEG is an unbelievably complex and nonlinear signal. The MindWave is able to use the onboard chip ThinkGear ASIC Module (TGAM1), with algorithms which reduce the noise and objects on the background. The TGAM1 chip features an algorithm for decomposing signals using the fast Fourier transform (FFT).

For classification of our four grouped MBTI personality traits, the features extracted using FFT are used by the HGP model.  Section 3 provides the details.

### 2.2. GP Life Cycle

GP [32–34] is indeed an evolutionary technique that is used to create a population of programmes that can be utilised to solve a problem by optimising them. The Darwinian theory, which gives the best chance of survival, is responsible for this creation [35, 36]. Koza [37] has been formalized and built into a functional method to pick the right approach from a huge variety of evolutionary techniques. GP is a heuristical and modular method that makes the representation by trees and graphs of complex systems that promote the handling of specific operations [38, 39]. Every individual is depicted to be a tree within the population. Tree representation includes function set and a terminal set that are unique for a particular problem. Lifecycle of GP consists of the following four steps as shown in Algorithm 1.

One of the key operators of GP for generating the solution is the crossover operator. The tendency of disruptive nature is the disadvantage of standard crossover operator. They may produce offspring having less fitness than their parents rather than good offsprings [21, 40, 41]. As a result, GP takes longer to reach the desired solution. Hybrid crossover [42–44] operator is also used in this paper instead of a regular crossover, which allows us to find the solution more efficiently and quickly. The details of hybrid crossover and the personality prediction model are given next.

## 3. Method Overview

This particular section discusses the methods used to apply our prediction model for personalities. It is split into two essential fields, where the experimental setup is clarified first and then the hybrid genetic programming for personality prediction is often described.

### 3.1. Experimental Setup

This section contains information about the participant pool, the device used for the experiment, the dataset utilised for the experiment, and finally the protocol for conducting the experiment.

#### 3.1.1. Pool of Participants

This study consists of 55 participants. However, from the final assessment 5 samples have been removed owing to hardware error or inappropriate EEG signals artifacts. Therefore, there are 50 representative samples of 18 to 46 years of age (38 males and 12 females). Tobacco and caffeine consumption was prohibited for 24 hours before to the study.

#### 3.1.2. Device Description

**Figure 2:**
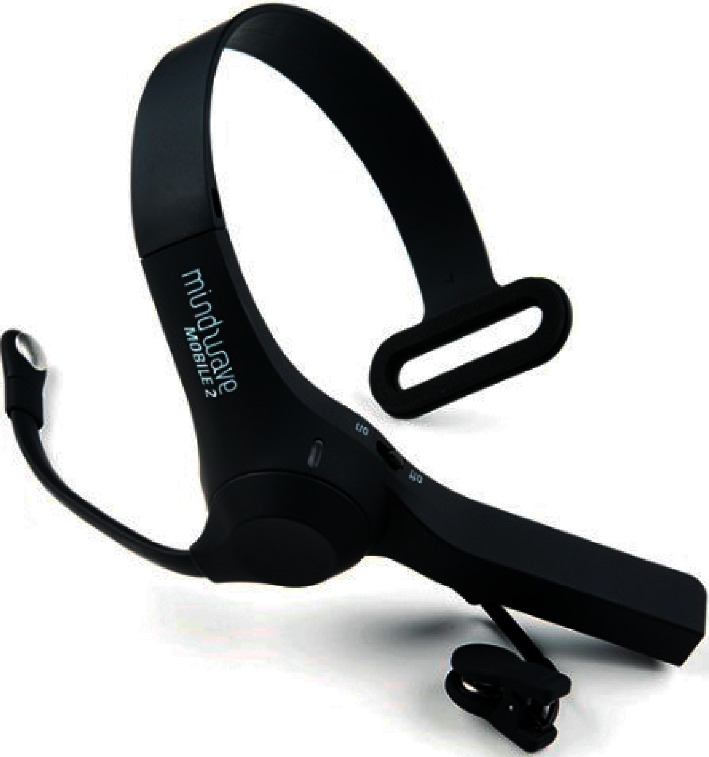
Single-channel Neurosky Mindwave Mobile 2.

The NeuroSky MindWave Mobile 2 [45] is a portable, easy-to-use EEG device whose functionality is to capture brain signals as seen in Figure 2. The brain wave-reading EEG headgear is easy to use and inexpensive. Physical components include flexible rubber sensor arms, a rounded forehead sensor tip, a T-shaped headband, and ear-clip contacts. The headset's binding electrodes are on the ear clip, and the EEG is on the sensor back, which is in front of the eye (FP1 position). The TGAM1 module is included in the package. It generates 12-bit (3–100 Hz) raw brainwaves at a rate of 512 Hz and generates EEG power spectrums in various frequency and morphological bands. This value is used for pairings with a Static Headset ID.

#### 3.1.3. Experimental Procedure

**Figure 3:**
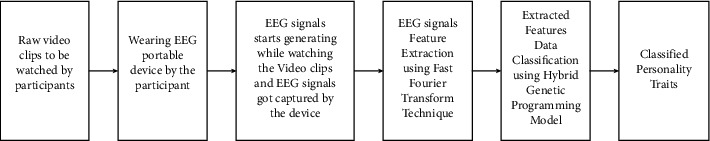
Personality prediction framework.

Every participant was made relaxed when they wear the EEG device. The method to construct the brain signal EEG dataset is described in Figure 3. This method is iterated 8 times during the training period with one participant. A starting hint of 10 seconds is given to the participant before the beginning of the test following that the participant viewed the video clips of a targeted personality trait. After watching each video clip, participants were required to fill the Likert scale of “agree,” “neutral,” or “disagree” self-evaluation form to determine the impact of each person's self-reported personality trait. It is composed of 4 grouped MBTI personality trait states mentioned earlier, and each group personality trait is in versus of each other. Participants were instructed to fill out the questionnaire based on their real thoughts while watching a film clip, rather than general emotions or attitudes. In each clip, a 2-minute buffer is provided with a neutral clip to monitor the effects of staring at the clip in the participant's head.

After all the questions of 4 grouped personality traits (for example, extraversion and introversion) are answered, we will evaluate the answers of each trait of the participant.

At the end of evaluation process of each trait, the trait having the highest counter value is labeled in the dataset. This marking scheme is taken as the ground truth for labeling EEG signals. Using this method, we will train our model. Four video clips will be used to collect the studies assessment data, each focusing on one personality trait from each group. Raw EEG signals are produced in each clip seen. Ten features related to FFT are part of the raw signals obtained by NeuroSky MindWave 2. With the help of all these features, each classifier will generate one output of the personality trait with whom the EEG signals will be matched from the trained dataset. In this way, from four classifiers, we will get four output personality traits. The combination of all the outputs is the final personality of the participant, and in this way, we will predict the participant personality.

### 3.2. Hybrid Genetic Programming for Personality Prediction

After generating the initial population of trees and calculating its fitness, genetic operators are applied on the individuals. Further portion is the complete explanation of hybrid crossover. The parameter values of the genetic operators are taken from Bhardwaj et al. [46].

#### 3.2.1. Hybrid Crossover

Once the fittest Nr individuals are transferred to the upcoming generation, i.e., reproduction operator of the HGP is applied on the individuals, later on, Nc remaining individual, the hybrid crossover get applicable. The hybrid crossover operator is a combination of the standard crossover and the constructive crossover operator. In this operator, the population of crossover is split into equal half (Nc/2). The standard crossover operator is used for the first half of the divided population and on the other half of split population. A hill-climbing technique for crossover is applied to the other half of the split population. The two newly generated offsprings are passed immediately to the upcoming generation in the standard crossover. The measures for the standard crossover as illustrated as follows:

- Two individuals out of the remaining population are randomly chosen as parents.
- Any random node is chosen from parent 1, and the entire subtree of that node is selected. Similarly, any random node is chosen from parent 2, and the entire subtree of that node is also selected.
- The selected subtree of parent 1 will get replaced by the selected subtree of parent 2 and vice-versa. In this way, two new offsprings are generated.

In hill climbing, the similar steps are taken for the generation of new offsprings as performed in standard crossover. For entering the upcoming generation, the conditions for individuals are different and they are as follows:

- All offspring are transmitted to the next generation if the fitness value of the two newly formed offsprings is greater than that of their parents. If even one offspring has a better fitness level than the parent, send it along with the parent to the next generation of fitter individuals.
- If both offsprings are less fit than their parents, crossover function is implemented recursively before one of the above two criteria is met. This recursion can be repeated up to ten times. If the fitness of the children is still insufficient to overcome the fitness of the parents, the parents are passed directly into the next generation.

At the end, the standard mutation operator [47] is applied on the lower *Nm* individuals.

## 4. Results and Discussions

This section discusses the findings for EEG signal study of the hybrid genetic programming operators. The Python (3.6) environment for implementation and the Intel I7 10^th^ gen laptop of 4.5 GHz with 16 GB of RAM is used for computation of hybrid genetic programming (HGP) classifiers. The FFT feature extraction method is used to extract important features from all the classifiers.

This study also included an assessment of the accuracy and confusion matrix of the existing and abovementioned current models.  Table 1 shows how the training and testing sets for EEG signal classification are divided using the 10-fold validation technique.

### 4.1. Experimental Results

Table 2 gives details about the testing set partition for classification of EEG signals into each personality trait.

Tables 3–6 give details about the confusion matrix of HGP1, HGP2, HGP3, and HGP4 classifiers, respectively. Our findings indicate that our model can accurately distinguish all classes, demonstrating the performance of our model.

Table 7 does a comparison of minimum accuracy (%), average accuracy (%), and maximum accuracy (%) of our work over the 10-fold cross-validation technique. There are 4 hybrid genetic programming classifiers, and they are termed as HGP1, HGP2, HGP3, and HGP4. For 10-fold partition, our classifier HGP1, HGP2, HGP3, and HGP4 achieved the average classification accuracy 79.166%, 80.95%, 80.242%, 79.295%, respectively. The minimum classification accuracy of HGP1, HGP2, HGP3, and HGP4 classifier is 77.21%, 78.45%, 78.32%, and 77.67%, respectively. Lastly, for 10-fold cross-validation our implemented classifier HGP1, HGP2, HGP3, and HGP4 achieved the maximum classification accuracy 81.86%, 82.74%, 82.68%, and 81.74%, respectively.  Table 8 shows the sensitivity, precision, and specificity values of HGP1, HGP2, HGP3, and HGP4 classifiers.

### 4.2. Comparison with Other Methods

Comparison of our implemented classifiers and other literature work is performed.  Table 9 shows that our implemented classifiers perform much better than other approaches in terms of classification accuracy. The authors of these studies have not stated whether the findings represent the maximum accuracy achieved by their classification approach or how the data are divided into training and testing partitions. Our implemented classifiers HGP1, HGP2, HGP3, and HGP4 are having the maximum classification accuracy as 81.86%, 82.74%, 82.68%, and 81.74% and the overall classification accuracy of the HGP classifier is 82.25% for 10-fold partition, which is calculated by taking out the mean of all the HGP classifiers accuracy. These results indicate the reliability of all the implemented HGP classifiers, and they are able to classify personality traits using the brain signals. Therefore, our experimental results reveal that the combination of FFT and the HGP is efficient means of identifying personality traits.

## 5. Conclusion, Limitations, and Future Work

A database of 30 Hindi and English language film clips is produced as part of this study. Also, an EEG-based personality prediction model was developed to aid in the identification of personality features in any individual. The relevant features are extracted using the fast Fourier transform approach, and then hybrid genetic programming is employed to classify personality traits. The HGP2 classifier got the best classification accuracy of 82.74% among four HGP classifiers, and the overall HGP classifier accuracy is 82.25%.

Fifty people took part and watched the film clips that targeted eight distinct personality traits. Such results revealed a gain in accuracy and possibility to identify personality traits over the existing state-of-the-art personality predictor systems. NeuroSky MindWave Mobile 2 device is used in this study to capture brain signals.

In addition, further audiences will be included in data collection and the impact of videos on various age ranges will also be evaluated. Currently, we plan to expand a single-channel device to multichannel device in the future [56, 57].

## Figures and Tables

**Algorithm 1:**
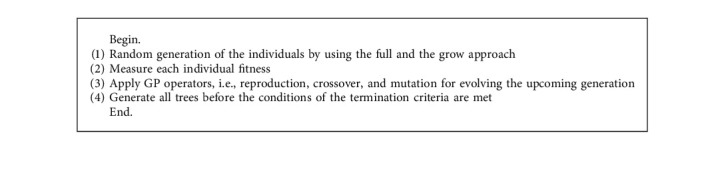
Genetic programming Algorithm.

**Table 1:** Partition of training and testing set for EEG signal classification on 10-fold validation schemes.

Training Testing	Total Samples	
	Number of training samples	Number of testing samples
10-fold split	16650	1850

**Table 2:** Partition scheme for testing set for EEG signals classification for each personality trait.

Training testing				Number of samples in the set				
	Extraversion	Introversion	Thinking	Feeling	Sensitive	Intuitive	Judging	Perceiving
10-fold split	280	200	251	245	210	210	200	254

**Table 3:** HGP1 classifier confusion matrix for extraversion vs introversion classification.

	Extraversion	Introversion
Extraversion	240	43
Introversion	57	140

**Table 4:** HGP2 classifier confusion matrix for thinking vs feeling classification.

	Thinking	Feeling
Thinking	228	43
Feeling	55	170

**Table 5:** HGP3 classifier confusion matrix for sensing vs intuition classification.

	Sensing	Intuition
Sensing	184	34
Intuition	46	156

**Table 6:** HGP4 classifier confusion matrix for judging vs perceiving classification.

	Judging	Perceiving
Judging	170	51
Perceiving	43	190

**Table 7:** Comparison of minimum accuracy (%), average accuracy, and maximum accuracy (%) of our work over 10-fold cross-validation technique.

Classifier	HGP1	HGP2	HGP3	HGP4
Average accuracy	79.166	80.95	80.242	79.295
Minimum accuracy	77.21	78.45	78.32	77.67
Maximum accuracy	81.86	82.74	82.68	81.74

**Table 8:** Comparison of performance measures.

Classifier	Sensitivity (%)	Precision (%)	Specificity (%)
	Mean ± std	Mean ± std	Mean ± std
HGP1	81.64 ± 2.48	80.25 ± 2.52	79.08 ± 2.44
HGP2	83.06 ± 2.14	82.14 ± 2.26	80.78 ± 2.18
HGP3	82.35 ± 2.62	81.57 ± 2.48	80.16 ± 2.52
HGP4	81.38 ± 2.32	80.47 ± 2.48	79.23 ± 2.42

**Table 9:** Classification accuracy comparison for personality prediction.

Author year	Method	Classification accuracy (%)
Tandera et al. [31]	LSTM + CNN	74.17
Tadesse et al. [48]	XGBoost	74.2
Mohammadi and Vinciarelli [49]	SVM	70
Pratama and Sarno [50]	Naive Bayes	60
Peng et al. [51]	SVM	73.50
Zhao et al. [52]	SVM	81.08
Moreno et al. [53]	LDA + LSVC	73
Ong et al. [54]	SVM	76.23
Shen et al. [55]	SVM	72
This study	HGP	82.25

## Data Availability

The data are available on request from the corresponding author.
